# Hints to the Junior Army and Navy Practitioners

**Published:** 1817-12

**Authors:** W. Robertson


THE LONDON

### Medical and Physical Journal.

6 OF VOL. XXXVIII.] DECEMBER, 1817. [NO. 226.

"For many fortunate discoveries in medicine, and for the detection of nume-
"rous errors, the world is indebted to the rapid circulation of Monthly
"Journals; and there never existed any work to which the Faculty in
"EUROPE and AMERICA were under deeper obligations than to the
"Medical and Physical Journal of London, now forming a long, but an
"invaluable, series."—RUSH.

*For the London Medical and Physical Journal.*

### *Hints to the Junior Army and Navy Practitioners;* by W. ROBERTSON, M. D.

"Differe quoque pro natura locorum, genera medicinæ; et aliud opus esse Romæ, aliud in Ægypto, aliud in Gallia."—*Celsus, de Med.*

"L'avantage extrême ou même la necessitè indispensable des descriptions topographique, a etè toujours vivement senti pour servir de base a l'histoire des maladies regnantes dans un lieu determinè."

"Il est sujet (l'homme) à tous les symptomes qui tiennent du charactere particulier de sa maladie, et il est modifie par la position des lieux, la nature du climat, les saisons, la maniere de vivre, les affections morales, dont il s'est formè longue habitude."—*Pinel, Medicine Clinique.*

I HAVE often lamented, Messrs. Editors, that, notwithstanding our numerous positions and settlements in the Mediterranean, no large and well-digested volume of Medical Facts and Observations has appeared relative to these countries, and more particularly their Medical Topography,—an interesting science, little cultivated by British physicians. Except the admirable work of Cleghorn, now of an old date, and the more recent spare one of the intelligent and lamented Irvine, we have no scientific work, that I am acquainted with, which embraces the double (but intimately connected) object of elucidating, by a correct history, the diseases of the climates of the Mediterranean, and (by a topographical development) an exposition of the causes, moral as well as physical, which are constantly operating in the production of all that diversity of phenomena which occur in the diseases of those latitudes, compared with similar affections of our own island. This must prove a subject of doubt, difficulty, and danger, to the physician, on first entering on his duties in these countries.

Does this deficiency of information arise from diffidence on the part of our army and navy practitioners? Afraid, perhaps, perhaps, of the shafts of a severe criticism, which, in our times, are sure, in every department of science, to accompany the footsteps of him who devotes his hours to the arduous task of instructing others, and enters the pale of the literary world; or, what I rather suspect to be the truth, the young practitioner is perplexed and involved in doubts, in regard to the *manner* in which he ought to proceed in drawing up his histories, and arranging his materials, for public inspection. Perhaps the following observations may tend to assist the tyro, stimulate his exertions, and induce him to attend to the subject of medical topography.

Sir John Pringle, in his excellent work "on the Diseases of the Army," observes, "In composing from my notes, I was long in doubt how to proceed—whether wholly to omit such things as are commonly known, or to treat all the diseases mentioned there in a full and regular manner." He then informs us that he determined to pass cursorily over those diseases common to Britain, while those of other climates he "handles at length." This advice may, I conceive, be attended to with much propriety, for, though some diseases may, in consequence, be described which are quite familiar to the physician in England, and are, perhaps, not greatly diversified by occurring in a warmer latitude, yet the object should be, the formation of a general work, for the use of those physicians and surgeons who may be employed in the Mediterranean. This work should be composed from copious notes, taken either at the bed-side of the patient, or immediately after the visit. The different authors on those diseases which the writer is to elucidate should be consulted, the opinions of his medical brethren canvassed, and, finally, the sentiments and opinions of native practitioners attended to. From the latter he will often receive much valuable information, particularly in what relates to the physical and moral states of the inhabitants, and of the agency of these conditions on the causes of disease. Hypotheses, which so frequently lead to error, (it is to be recollected that the writer is addressing the tyro,) should be sedulously avoided; for to no science does the observation of Voltaire more aptly apply than to that of medicine. "Il y a un nombre innombrable de manieres d'arriver á l'erreur, il n'y a qu'une seule route vers la verité ; il y a donc l'infini contre un à parier, qu'un philosophe qui ne s'appui era que sur des hypotheses, ne dira que dès chimeres." *Elem. de la Philos.*

Every improvement in natural knowledge must be derived from careful observation and experiment. Cicero observes, "Opinionum commenta delet dies, naturæ judicia confirmat;"

firmat;" and my Lord Bacon, "Intermissio diligentiæ illius Hippocratis, utilis admodum et accuratæ, cui moris erat narrationem componere casuum circa ægrotos specialium; referendo, qualis fuisset morbis natura, qualis medicatio, qualis eventus." "Quis autem ad observandum, adjiciet animum, ei etiam, in rebus quæ vulgares videntur, multa observatu digna occurrent." *De Augm. scientarum.* "To know facts, to separate them from suppositions, to arrange and connect them, and, above all, to study their useful applications," should form the object of ambition of the young physician. While we thus proscribe the allurements and fascinations of hypothesis, we strongly inculcate in their place the study of those sciences connected with medicine. "Ista rerum contemplatio, quamvis non faciat medicum, aptiorem tamen medicinæ reddit." *Celsus.*

In regard to that department of the work which is to embrace medical topography, it may be adviseable to divest it of all technical and general phraseology, as much as the subject will admit, that it may be acceptable to men of general science.

In dedicating a large portion of his work to physical topography, an author may possibly incur the censure of the English resident physician; but it is to be recollected that he writes principally for men who are about to enter on climates, seasons, manners, and customs, totally different from those they have been accustomed to, and which have a wonderful influence in modifying the appearances of disease. This study ought assuredly, therefore, to arrest the consideration of the pathologist, not only as aiding in the developement of the causes of diseases, so frequently obscure, and of pointing out the action of different moral and physical agents on the living system, but also as the foundation on which any preventive measures can, with a probability of success, be erected. The ancients regarded this knowledge as essential to the physician; and Hippocrates constantly refers to it in his works.

If, in Britain, too little attention has been paid to medical topography, the converse of the position holds in regard to the French nation: certainly much extraneous and florid description enters into their detailed cases; yet we must acknowledge that additional interest is derived from that source. In proof, we need only mention the enthusiasm and lively interest which every student must feel in tracing the topography of Sicily and other islands in the Mediterranean, and, most of all, Egypt. On the far-extending shores of the Mediterranean, the eye rests with a peculiar delight, placid as its unruffled waters; while the mind, in pleasing

 reverie, reverie, retraces, in long succession, its eloquent statesmen, its profound and venerable authors, its renowned warriors, its ancient kings, its exquisitely sculptured temples, its vast and stupendous pyramids, the wonder of our world;* and, though last, assuredly the most sublime, the meek Author of our holy religion. To these coasts the sciences, "which, of all the possessions of man, seem least to partake of the imperfection of his nature,"† and he himself, refer their origin. Here immutable demonstrations of the perfection to which the arts had attained in ages beyond the records of men still exist, in multiplied profusion, to attest the early polity, wisdom, and splendour, of their former inhabitants.

> *Τω παρα ναιεταουσιν αριπρεπεων γενος ανδρων,*
> *Οι πρωτοι βιοτοιο διεστησαντο κελευθους,*
> *Πρωτοι δ' ιμεροεντος επειρησαντο αροτρου,*
> *Και σπορον ιθυτατης υπερ αυλακος απλωσαντο·*
> *Πρωτοι δε γραμμησι πολον διεμετρησαντο,*
> *Θυμω φρασσαμενοι λοξον δρομον ηελιοιο.*
> * * * * *
> *Ἠ μεν οσοι Θηβην, ερικυδεα ναιεταεσκον,*
> *Θηβην ωγυγιην, εκατομπυλον, ενθα γεγωνως*
> *Μεμνων αντελλουσαν εην ησπαζετο Ηω.*
>
> Διονυσιου ΟΙΚΟΥΜΕΝΗΣ ΠΕΡΙΗΓΗΣΙΣ, p. 41, 45.

While evidence is thus afforded of a superior race of people, how does the heart sicken at beholding these fertile regions immersed in worse than Gothic barbarism, under a government which language cannot find terms adequately vile to pourtray!

It may be urged, that an enlarged detail, such as is here proposed to be given, on the subject of medical topography, is irrelevant to the subject, and foreign to the labours of the physician, who, in a treatise on the diseases of a particular district or country, ought to compress this department into as small a compass as may be. To this objection it may be observed, that an extended view can alone afford to strangers that species of information which they are most in want of, which is the least easily procured at a time when it is most required, and, it may be remarked, which is not attainable in the English language, or, indeed, from almost any written source, for reasons already assigned.

---

* A late author observes, that the ideas associated on the first view of the Pyramids are those "of duration, almost endless; of power, inconceivable; of majesty, supreme; of solitude, most awful; of grandeur, of desolation, and of repose."—*Clarke's Travels*, part 2d, sect. 2d, p. 46.

† Dr. Currie.

This reasoning holds particularly in regard to Egypt. In our expedition to that fertile country in 1801, our officers, medical as well as military, could find no topographical treatise, in any language, relating to the invaded country; and it must be admitted, notwithstanding the acquisitions we have received from authors who have written the history of our descent on Egypt, that much *terra incognita* still remains.

Should the Ottoman empire, that mouldering mass of venality, corruption, and violence, meet the fate which is apparently suspended over it, England may again send her armies and navies to the shores of Egypt and the Archipelago, not to assist other powers, but to claim a portion of these beautiful but oppressed countries for herself, when a knowledge of their physical and medical topography, always of importance to the medical philosopher and pathologist, must find a more than usual share of public interest.

In a volume published at Paris in 1802, (Histoire Médicale de l'Armèe d'Orient, par le Medecin en chef B. Desgenettes,) a circular letter is addressed to the ordinary physicians of the French army, in which he (Desgenettes) engages them to collect all possible information relative to the physical and medical topography of the different situations in which they might be placed. This is now transcribed for the young physician's information, as an useful table. The plan of observations proposed is,

"1. To indicate the nature of the soil of the country to be described.

"2. The longitude, latitude, and general exposure.

"3. The prevailing winds.

"4. The principal physical qualities of the water of the Nile, of the wells, and of cisterns; their influence on vegetation, and on the health of man and domestic animals.

"5. The trees, shrubs, and other plants, particularly the culinary and medicinal.

"6. The grains which are cultivated; their manner of cultivation; the diseases to which they are liable.

"7. To examine with care, and to point out, the numerous medicinal substances which the commerce of Asia furnishes to Africa, and particularly to Egypt.

"8. Animals of all classes which are particular to Egypt; the diseases of those domestic animals which are most useful to man.

"9. To describe the general characters of the inhabitants; their food, drinks, clothing, construction of their dwellings; their occupations, customs, manners; the most common diseases incident to children, to the men, to the young and to the married women, and their common method of treatment; at what epoch the women begin and cease to menstruate; their fecundity; the usual term of life."

It is truly and deeply to be lamented, that, notwithstanding the numerous expeditions which our country has sent from its shores during these last 100 years, so very few of the physicians and surgeons have conceived it their duty, or felt it to be their ambition, to detail the medical history of these armaments. What a fund of useful knowledge has been lost to us and to future generations! To medicine, as an experimental art, every species of information, however distantly connected with it, is of importance; as it is only by an accumulation of facts and observations, through a long series of revolving years, that our science can hope to advance towards perfection; for, as Baglivi has truly observed, "Medicina non ingenii humani partus, sed temporis filia." May we hope, that a field so extensive and so little cultivated may arrest the consideration of those who may, in future, be intrusted with the medical department of our armies and navies; and that they may not be deterred, either by a dread of criticism, or by too great a diffidence in their own mental acquisitions, from undertaking a task which their country will expect of them,—a task, which, unless performed by some, the experience and knowledge of new facts and observations, which must be gained by numerous individuals, will again pass into oblivion. "Quemadmodum enim aqua, sive ex cælesti rore descendens, sive ex fontibus scaturiens, facile dispergitur et disperditur nisi colligatur in aliqua receptacula, ubi per unionen et congregationem se sustentare et fovere possit; similiter, liquor iste scientiæ pretiosissimus mox periret omnis atque evanesceret, nisi conservaretur in libris." *Bacon de Aug. Scientiarum.*

*Berwick-on-Tweed;*

*Sept.* 3, 1817.

---

*For the London Medical and Physical Journal.*

